# Amyloid-β colocalizes with apolipoprotein B in absorptive cells of the small intestine

**DOI:** 10.1186/1476-511X-8-46

**Published:** 2009-10-22

**Authors:** Susan Galloway, Ryusuke Takechi, Menuka MS Pallebage-Gamarallage, Satvinder S Dhaliwal, John CL Mamo

**Affiliations:** 1 The Australian Technology Network Centre for Metabolic Fitness, School of Public Health, Curtin Health Innovation Research Institute, Curtin University of Technology, Perth, Western Australia, Australia

## Abstract

**Background:**

Amyloid-β is recognized as the major constituent of senile plaque found in subjects with Alzheimer's disease. However, there is increasing evidence that in a physiological context amyloid-β may serve as regulating apolipoprotein, primarily of the triglyceride enriched lipoproteins. To consider this hypothesis further, this study utilized an in vivo immunological approach to explore in lipogenic tissue whether amyloid-β colocalizes with nascent triglyceride-rich lipoproteins.

**Results:**

In murine absorptive epithelial cells of the small intestine, amyloid-β had remarkable colocalization with chylomicrons (Manders overlap coefficient = 0.73 ± 0.03 (SEM)), the latter identified as immunoreactive apolipoprotein B. A diet enriched in saturated fats doubled the abundance of both amyloid-β and apo B and increased the overlap coefficient of the two proteins (0.87 ± 0.02). However, there was no evidence that abundance of the two proteins was interdependent within the enterocytes (Pearson's Coefficient < 0.02 ± 0.03), or in plasma (Pearson's Coefficient < 0.01).

**Conclusion:**

The findings of this study are consistent with the possibility that amyloid-β is secreted by enterocytes as an apolipoprotein component of chylomicrons. However, secretion of amyloid-β appears to be independent of chylomicron biogenesis.

## Background

Amyloid-β is recognized as the principal protein in senile plaques in subjects with Alzheimer's disease (AD) [1]. Generated from the slicing of amyloid precursor protein (βAPP) by secretases, the synthesis of amyloid-β can be differentially modulated by cellular lipid homeostasis. Studies in cell culture and in vivo suggest that cholesterol inhibits amyloid-β biogenesis [2-4], although this effect may be dependent on the distribution of free and esterified cholesterol within the plasma membrane and within lipid rafts [5]. In contrast, in vivo studies found that chronic ingestion of diets enriched in saturated-fats (SFA) had a potent stimulatory effect on enterocytic amyloid-β abundance [6].

Several lines of evidence suggest that one physiological role for amyloid-β is as a regulating apolipoprotein, particularly of the triglyceride-rich lipoproteins (TRL's). Koudinov et al reported that amyloid-β is secreted by hepatocytes as a lipoprotein complex [7]. Significant plasma abundance of amyloid-β was also found in the TRL fraction of control subjects and amyloid-β enrichment in TRL's was evident in subjects with AD, or with mild cognitive impairment [8]. Ingestion of a lipid rich meal also causes a transient increase in plasma of soluble APP, concomitant with postprandial lipaemia [9] and when injected intravenously associated with TRL-emulsions, amyloid-β increased uptake in fat-rich tissues relative to liver [10].

The βAPP is expressed on the plasma membrane of a number of tissues including lipogenic organs such as liver [6]. Proteolytic cleavage of βAPP generally results in the extracellular release of amyloid-β which is then chaperoned by transporter proteins [11-14]. However, hydrophobic domains within amyloid-β [15] results in rapid folding of amyloid-β that make it unlikely to readily associate with lipoproteins already secreted into circulation. Rather, immunhistochemistical studies show amyloid-β abundance within the perinuclear region of hepatocytes and absorptive epithelial cells of the small intestine [4,6,7,16], suggesting that amyloid-β may form part of the primordial lipoprotein during the lipidation process. Consistent with the latter, using a phage display Nelson and Alkon showed that amyloid-β bound tightly with several apolipoproteins found commonly with TRL [17]. To further consider the possibility that amyloid-β becomes associated with nascent lipoproteins, in this study we utilized sensitive three-dimensional (3D) immunofluorescent (IF) microscopy to explore if enterocytic abundance of amyloid-β is associated with chylomicrons. Apolipoprotein (apo) B, an obligatory component of TRL secreted by intestine and liver, was used as a marker of enterocytic chylomicron distribution and plasma abundance of TRL.

## Materials and methods

### Diet and animals

The protocols described in this study were approved by an accredited National Health and Medical Research Council of Australia Animal Ethics Committee (Curtin University Animal Experimentation and Ethics Committee Reference number R02-07). Six-week-old female C57BL/6J mice (Animal resources centre, Murdoch, Western Australia) were divided randomly into a low-fat (LF) or saturated fatty acid (SFA) diet group. Low fat mice were given chow that contained 3.6% (w/w) as unsaturated fat and 0.4% SFA (AIN93M, Specialty Feeds, Western Australia). The SFA enriched chow contained 12.9% (w/w) as saturated fats and 7.4% as unsaturated oils (SF07-50, Specialty feeds, Western Australia). Both diets were free of cholesterol. Digestible energy for LF and SFA feed were 15.1 MJ/kg and 18.8 MJ/kg respectively and feed was available *ad libitum*. After three-months of dietary intervention, mice were sacrificed by pentobarbital injection. The small intestine was isolated and flushed with chilled phosphate buffered saline (PBS, pH 7.4). A 2 cm segment of the small intestine distal to the duodenum was fixed in 4% paraformaldehyde for a minimum of 24 h, processed and longitudinal segments embedded in paraffin wax. Serial sections of 5 μm thick were cut on microtome and mounted on silanised slides for histology and immunofluorescence microscopy.

### Antibodies

Anti-apo B, anti Golgi-apparatus (anti-Golgi 58 K), anti-rabbit IgG with Alexa488, and streptavidin-Alexa546 were obtained from Invitrogen (Melbourne, Victoria, Australia). Anti-rabbit IgG biotin conjugate was obtained from DAKO (Glostrup, Denmark). Rabbit anti-human amyloid-β was obtained from Chemicon International (Temecula, California, United States).

#### Double-immunofluorescent labelling

An established double IF labelling method was utilized as previously described [18]. Cross reactivity was prevented using a biotin-avidin amplification technique microscopy. The concentration of the primary antibody used with biotin-avidin amplification is substantially below the threshold required for detection by standard IF and does not interfere with detection of the second protein.

Anti-amyloid-β (1:1000) was added to sections overnight at 4°C, followed by addition of goat anti-rabbit IgG with biotin (1:200) for 1 h at room temperature. Thereafter, anti-Golgi-apparatus (1:10) was added overnight at 4°C. Immunofluorescence was detected by streptavidin-Alexa546 (1:100) and anti-rabbit IgG with Alexa488 (1:100) for amyloid-β and Golgi-apparatus respectively. Cell nuclei were detected using DAPI and slides were mounted using anti-fade mounting medium. The same method was used to achieve double apo B and Golgi-apparatus staining by substituting the anti-amyloid-β with anti-apo B (1:400).

### Image capture

Digital images were captured using AxioCam mRM and ApoTome on a Zeiss Axiovert 200 M inverted microscope and visualized with Plan-NeoFluar lenses (Carl Zeiss, Oberkochen, Germany). Excitation and emission were achieved by using filters 43 (Ex BP545/25, beam splitter FT570 and Em BP605/70) and 38 (Ex BP470/40, beam splitter FT495 and Em BP525/50) to determine fluorescence of Alexa546 and Alexa488 respectively. Filter 49 (Ex G365, beam splitter FT 395 and Em BP445/50) was used to detect nuclei stain DAPI. Individual channels are devoid of fluorescence from other emission sources and are therefore clear of bleed-through.

Three-dimensional images were captured using the ApoTome optical sectioning mode which allows the creation of a 3D image based on the 'stacking' of consecutive 2D images. Each 3D image consisted from 8-10 2D images, and the axial distance of Z-stack was 0.5 μm for 200×. There were 6 animals per group with a minimum of 40 images per mouse used for analysis. Fluorescent intensity and area were determined using the measurement and colocalization module available on AxioVision v4.7.1 software (Carl Zeiss, Oberkochen, Germany).

### Quantification of fluorescent intensity and colocalization

There are several algorithms capable of achieving measures of colocalization or association via measurement of fluorescent pixel spatial orientation and pixel intensity. The Pearson's correlation coefficient (r) is a commonly used quantitative estimate of association (abundance) for proteins [19]. However, as Pearson's correlation is a measure of variance from the mean pixel intensity, it does not provide information of the area of overlap. A modification to Pearson's correlation coefficient developed by Manders et al (1993) eliminates the average grey values from the Pearson's formula to allow the quantification of overlapping pixels from each channel [19]. The degree of colocalization for the proteins is positively related to the Manders coefficient, known commonly as the 'overlap coefficient' (OC). The AxioVision software utilizes an automated procedure based on spatial statistics to determine Pearson's correlation coefficient and Manders OC, thereby avoiding selection bias by manual selection methods.

### Western blotting for plasma apolipoprotein B

Plasma samples were separated on NuPAGE 3-8% Tris-acetate gels (EA03752BOX, Invitrogen, Victoria, Australia) at 150 V (Biorad Model 20012.0) for 1 hr. Gels were then electrotransferred to PVDF membranes (PV4HY00010, Osmonics Inc, Minnesota U.S.A) at 40 V for 1 hr and blocked in 10% skim milk (in TBST) overnight at 4°C. The membranes were incubated with polyclonal rabbit anti-human apo B 1:100 (Q0497, Dakocytomation, Glostrup, Denmark), and then with donkey anti-rabbit immunoglobulin G (IgG) horseradish peroxidase (HRP) (Na934V, Amersham Bioscience, Buckinghamshire, UK). Proteins were detected using enhanced chemiluminescence reagent (ECL™) western blotting analysis system (RPN2108, Amersham Bioscience, Buckinghamshire, UK). Membranes were exposed to high performance chemiluminescence film (Amersham Hyperfilm™, Amersham Bioscience, Buckinghamshire, UK) and developed in an AGFA-Gevaert Rapidoprint X-Ray Developer (Septestraat, Belgium). Apo B48 bands were identified and quantified by densitometry against purified apo B48 protein of known mass (550 kDa for apo B-100 and 260 kDa for apo B48).

### Amyloid-beta ELISA

Plasma amyloid-β 40/42 levels were measured using commercially available ELISA kits (Biosource, Camarillo CA).

### Statistics

Enterocytic colocation of amyloid-β with apo B was determined by an automated procedure based on spatial statistics to determine Pearson's correlation coefficient and Manders OC (AxioVision 4.0). The association between total apo B, apo B48, and apo B100 with total amyloid-β, amyloid-β40 and amyloid-β42 were examined using Pearson's and Spearman's correlation. Spearman's correlation was used when the assumptions of the analysis were violated due to the presence of outliers. P-values less than 5% were considered as statistically significant and the data was analysed using SPSS version 17.0.

## Results

**Figure 1:**
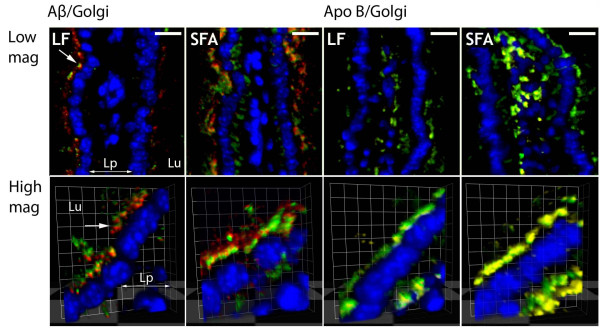
**Enterocytic amyloid-β and apolipoprotein B colocalizes with Golgi-apparatus under LF and SFA feeding**. The images depict the colocalization of Golgi-apparatus with amyloid-β (columns 1 and 2) and apo B (columns 3 and 4) in low-fat (LF) and saturated fat (SFA) fed mice. The upper row shows small intestinal villi at low magnification (mag) in two dimension, whilst the lower frames depicts enterocytes at high magnification in three dimensions. Amyloid-β as indicated in red, apo B as yellow, Golgi-apparatus as green, and nuclei as blue pixels. Where overlap of pixels occurs between amyloid-β (red) and Golgi-apparatus (green), an orange colour prevails. Similarly, the colocalization of apo B (yellow) with Golgi-apparatus (green) generates lime colour. Perinuclear (white arrow) and lamina propria (Lp) presence of amyloid-β and respective proteins are shown. Lu labels the lumen that represents the apical surface of the cell and Lp (lamina propria) is the direction of lacteals where lipoproteins are expelled via exocytosis. Scale: bar (2D images) = 10 μm; grid (3D images) = 3.63 μm.

**Figure 2:**
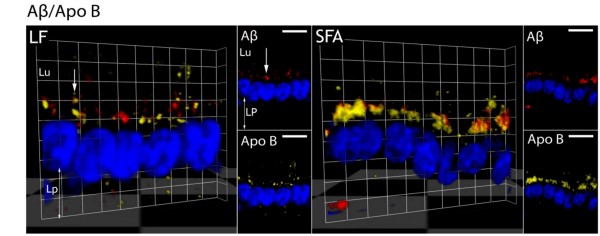
**Enterocytic colocalization of amyloid-β with apo B under LF and SFA feeding**. The enterocytic colocalization of amyloid-β (Aβ) with apolipoprotein B (apo B) in low-fat (LF) and saturated fat (SFA) fed mice is shown in three dimensions. The inset images depict the separate channel view for Aβ and apo B respectively. Amyloid-β is seen in red pixels, apo B as yellow and nuclei as blue. The perinuclear region (white arrow) and lacteal (Lp) orientation of enterocytes is indicated. Lu labels the lumen that represents the apical surface of the cell and Lp (lamina propria) is the direction of lacteals where lipoproteins are expelled via exocytosis. Scale: bar (2D inset images) = 10 μm; grid (3D images) = 3.63 μm.

Enterocytic chylomicrons were detected by determining the distribution of apo B, an obligatory structural component of chylomicrons. Significant amounts of amyloid-β and apo B were found to be enriched within the perinuclear region of cells. Amyloid-β and apo B colocalized with the Golgi-apparatus, towards the basolateral surface of the cell and within the lacteals (Figure 1). The patterns of distribution for amyloid-β and apo B remained essentially the same in LF and SFA fed mice (Figure 2), however abundance of each protein more than doubled in SFA fed mice compared to LF fed animals (Table 1, columns 1 and 2).

**Table 1:** Effect of SFA feeding on concentration and colocalization of enterocytic amyloid-β with apo B.

	Apo B*		Amyloid-β*		**Overlap Coefficient**		**Pearson's Coefficient**	
	
	**Mean**	**SEM**	**Mean**	**SEM**	**Mean**	**SEM**	**Mean**	**SEM**
**LF**	7013	790	5403	404	0.730	0.033	0.020	0.027
**SFA**	15840^	1812	13224^	1002	0.872^	0.022	0.015	0.023

*Mean enterocytic pixels value is expressed as mean densitometric sum and standard error of mean (SEM).

^Statistical significance was observed between LF and SFA groups with a p-value of at least less than 5%.

The colocalization of enterocytic amyloid-β and apo B was expressed as the OC (Manders overlap coefficient). The relative abundance of amyloid-β and apo B in LF and in SFA fed mice, given as mean densitometric sum. In LF mice, approximately 73% of immunodetectable amyloid-β colocated with apo B, but in SFA mice this was significantly increased (*p *< 0.05) to nearly 87% (Table 1, columns 3 and 4). Figure 2 shows the extent of colocalisation in three dimensions of amyloid-β relative to apo B under high magnification.

**Figure 3:**
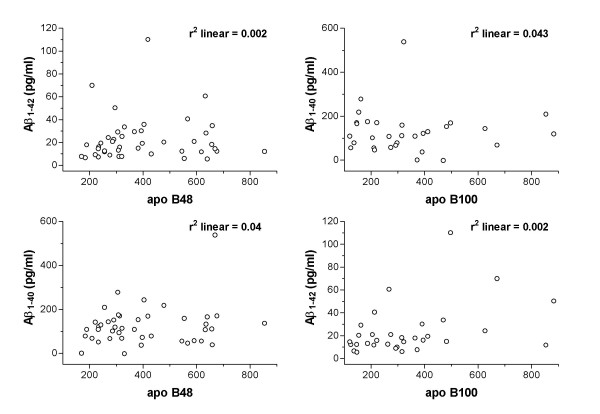
**Correlation analysis of plasma amyloid-β40/42 with plasma apo B48/100**. Correlation coefficients were determined with Pearson's correlation analysis where no outliers were identified.

To explore if abundance of the amyloid-β was inter-dependent with TRL biogenesis and secretion, correlation analysis with apo B was determined within enterocytes and in plasma respectively. Pearson's correlation analysis found that just 2% of amyloid-β and apo B fluorescent intensities were positively associated in enterocytes of LF or any of the SFA fed mice (table 1). Similarly, in plasma there was no evidence that the principal isoforms of amyloid-β (amyloid-β40 and 42) were associated with intestinal or hepatic apo B lipoproteins (figure 3).

## Discussion

In this study the distribution and abundance of amyloid-β and apo B were detected in small intestinal enterocytes using an established double-labeled avidin-biotin IF microscopy technique [18]. Amyloid-β and chylomicron-apo B were remarkably colocalized in enterocytes, consistent with release of amyloid-β as a lipoprotein complex [7]. We also confirm that chronic consumption of SFA increases enterocytic amyloid-β and now show that this occurrence is concomitant with a substantially greater abundance of enterocytic apo B [6]. However, there was no evidence from this study that the biogenesis of amyloid-β and apo B are inter-dependent based on Pearson's correlation analysis within enterocytes and in plasma.

The biosynthesis of chylomicrons occurs in a multi-step process that requires the progressive lipidation of apo B an obligatory structural component of primordial lipoproteins secreted by the small intestine [20,21]. A number of proteins are reported to associate with nascent chylomicrons prior to secretion, including apo A-I, A-IV, apo J, apo D, apo E and small molecular weight proteins such as apo C-II. Nascent chylomicrons are then transported via the Golgi-apparatus to the basolateral membrane and secreted into lymphatics. The results from this study suggest that amyloid-β is secreted from small intestinal enterocytes as an apolipoprotein of chylomicrons.

Immunoreactivity for amyloid-β and apo B was found selectively within the ER/Golgi-apparatus and not on the plasma membrane. The findings are consistent with biogenesis of amyloid-β at the ER and translocation to primordial lipoproteins, rather than as a consequence of β APP processing. Similar results in hepatocyte cultures with secretion of amyloid-β also occurring exclusively as a lipoprotein complex [7].

Dietary SFA promote chylomicron biogenesis by stimulating apo B lipidation [22,23], an essential step to avoid post-translational degradation by intracellular proteases [24]. Greater lipid substrate availability (as a result of SFA ingestion) reduces the proportion of apo B that would otherwise be degraded. The SFA dietary intervention used in this study essentially doubled enterocytic apo B and a similar increase in amyloid-β abundance was observed. However, the mechanisms by which SFA stimulate amyloid-β abundance and association with nascent chylomicrons are less clear. Saturated-FA may have a broader non-specific effect on enterocytic protein synthesis and consistent with the possibility of substrate driven biogenesis, Patil (2006) [25] found in neurons treated with palmitic acid resulted in increased upregulation BACE, a key enzyme complex involved in the processing of β *APP*. Alternatively, amyloid-β is an amphiphatic protein with a C-terminal domain that avidly binds with negatively charged hydrophobic lipids [15]. Increased substrate availability and synergistic lipidation of amyloid-β and apo B may promote the incorporation of amyloid-β into nascent chylomicrons and subsequently stimulate further synthesis of the proteins.

The SFA induction and secretion of enterocytic amyloid-β may be important in the context of AD risk. Recent studies suggest that blood-to-brain delivery of amyloid-β may contribute to amyloidosis, particularly when the concentration of circulating amyloid-β is chronically elevated [26-28]. This study suggest that SFA's increase synthesis and secretion of TRL associated amyloid-β concomitant with deterioration in blood-brain barrier integrity [29]. Indeed, the hypothesis is supported by studies in transgenic mice that over-express amyloid-β. In β APP/presenilin 1 transgenic mice, the plasma concentration correlated with secretion rates into blood of TRL's, which was increased 3-8 fold above wild-type mice [27]. Moreover, there was a positive association between plasma TRL-amyloid-β secretion with onset of cerebrovascular and parenchymal amyloidosis [29].

## Conclusion

In this study, evidence in vivo that amyloid-β is secreted as a chylomicron complex and is stimulated by dietary SFA's is presented. Exploring this phenomenon in the context of plasma amyloid-β homeostasis and lipoprotein kinetics may provide insight into the putative association of high-fat diet with AD risk.

## List of Abbreviations

AD: Alzheimer's disease; apo: apolipoprotein; βAPP: β-amyloid precursor protein; IF: immunofluorescence; LF: low-fat; OC: overlap coefficient; PBS: phosphate buffered saline; SFA: saturated-fatty-acid; TRL: triglyceride-rich-lipoprotein

## Competing interests

The authors acknowledge that there is no conflict of interest of any prior publication of any materials presented herein. All authors have seen and support the publication of this manuscript.

## Authors' contributions

SG carried out the design of project, collection of data, immunofluorescence, statistical analysis and drafting of the manuscript. RT and MP-G assisted in the collection of tissues, interpretation of data and critically analyzing the manuscript content. SD helped in the statistical analysis of data and critically analyzing the manuscript content. JM conceived the study, helped in the interpretation of data, drafting of the manuscript, acquiring funding and role in general supervision of the research group. All authors have approved submission of the manuscript.

## References

[B1] Glenner GG, Wong CW (1984). Alzheimer's disease: initial report of the purification and characterization of a novel cerebrovascular amyloid protein. Biochem Biophys Res Commun.

[B2] Park IH, Hwang EM, Hong HS, Boo JH, Oh SS, Lee J, Jung MW, Bang OY, Kim SU, Mook-Jung I (2003). Lovastatin enhances Abeta production and senile plaque deposition in female Tg2576 mice. Neurobiol Aging.

[B3] Runz H, Rietdorf J, Tomic I, de Bernard M, Beyreuther K, Pepperkok R, Hartmann T (2002). Inhibition of intracellular cholesterol transport alters presenilin localization and amyloid precursor protein processing in neuronal cells. J Neurosci.

[B4] Pallebage-Gamarallage MM, Galloway S, Johnsen R, Jian L, Dhaliwal S, Mamo JC (2009). The effect of exogenous cholesterol and lipid-modulating agents on enterocytic amyloid-beta abundance. Br J Nutr.

[B5] Simons K, Ikonen E (2000). How cells handle cholesterol. Science.

[B6] Galloway S, Jian L, Johnsen R, Chew S, Mamo JC (2007). beta-amyloid or its precursor protein is found in epithelial cells of the small intestine and is stimulated by high-fat feeding. J Nutr Biochem.

[B7] Koudinov AR, Koudinova NV (1997). Alzheimer's soluble amyloid beta protein is secreted by HepG2 cells as an apolipoprotein. Cell Biol Int.

[B8] Mamo JC, Jian L, James AP, Flicker L, Esselmann H, Wiltfang J (2008). Plasma lipoprotein beta-amyloid in subjects with Alzheimer's disease or mild cognitive impairment. Ann Clin Biochem.

[B9] Boyt AA, Taddei K, Hallmayer J, Mamo J, Helmerhorst E, Gandy SE, Martins RN (1999). Relationship between lipid metabolism and amyloid precursor protein and apolipoprotein E. Alzheimer's Reports.

[B10] James AP, Pal S, Gennat HC, Vine DF, Mamo JCL (2003). The incorporation and metabolism of amyloid-beta into chylomicron-like lipid emulsions. J Alzheimers Dis.

[B11] Golde TE, Eckman CB (2001). Cholesterol modulation as an emerging strategy for the treatment of Alzheimer's disease. Drug Discov Today.

[B12] Koudinov A, Matsubara E, Frangione B, Ghiso J (1994). The soluble form of Alzheimer's amyloid beta protein is complexed to high density lipoprotein 3 and very high density lipoprotein in normal human plasma. Biochem Biophys Res Commun.

[B13] Zlokovic BV, Martel CL, Mackic JB, Matsubara E, Wisniewski T, McComb JG, Frangione B, Ghiso J (1994). Brain uptake of circulating apolipoproteins J and E complexed to Alzheimer's amyloid beta. Biochem Biophys Res Commun.

[B14] LaDu MJ, Pederson TM, Frail DE, Reardon CA, Getz GS, Falduto MT (1995). Purification of apolipoprotein E attenuates isoform-specific binding to beta-amyloid. J Biol Chem.

[B15] Shao H, Jao S-C, Ma K, Zagoriski MG (1999). Solution Structures of Michelle-bound Amyloid beta1-40 and bet1-42 Peptides of Alzheimer's Disease. J Mol Biol.

[B16] Galloway S, Pallebage-Gamarallage MM, Takechi R, Jian L, Johnsen RD, Dhaliwal SS, Mamo JC (2008). Synergistic effects of high fat feeding and apolipoprotein E deletion on enterocytic amyloid-beta abundance. Lipids Health Dis.

[B17] Nelson TJ, Alkon DL (2007). Protection against beta-amyloid-induced apoptosis by peptides interacting with beta-amyloid. J Biol Chem.

[B18] Takechi R, Galloway S, Pallebage-Gamarallage MM, Johnsen RD, Mamo JC (2008). Three-dimensional immunofluorescent double labelling using polyclonal antibodies derived from the same species: enterocytic colocalization of chylomicrons with Golgi apparatus. Histochem Cell Biol.

[B19] Manders EMM, Verbeek FJ, Atenm JA (1993). Measurement of co-localization of objects in dual-colour confocal images. J Microsc.

[B20] Hussain MM, Kedees MH, Singh K, Athar H, Jamali NZ (2001). Signposts in the assembly of chylomicrons. Front Biosci.

[B21] van Greevenbroek MM, de Bruin TW (1998). Chylomicron synthesis by intestinal cells in vitro and in vivo. Atherosclerosis.

[B22] Davidson NO, Kollmer ME, Glickman RM (1986). Apolipoprotein B synthesis in rat small intestine: regulation by dietary triglyceride and biliary lipid. J Lipid Res.

[B23] Green PH, Riley JW (1981). Lipid absorption and intestinal lipoprotein formation. Aust N Z J Med.

[B24] Lee DM, Singh S (1988). Degradation of apolipoprotein B-100 in human chylomicrons. Biochim Biophys Acta.

[B25] Patil S, Sheng L, Masserang A, Chan C (2006). Palmitic acid-treated astrocytes induce BACE1 upregulation and accumulation of C-terminal fragment of APP in primary cortical neurons. Neurosci Lett.

[B26] LaRue B, Hogg E, Sagare A, Jovanovic S, Maness L, Maurer C, Deane R, Zlokovic BV (2004). Method for measurement of the blood-brain barrier permeability in the perfused mouse brain: application to amyloid-beta peptide in wild type and Alzheimer's Tg2576 mice. J Neurosci Methods.

[B27] Burgess BL, McIsaac SA, Naus KE, Chan JY, Tansley GH, Yang J, Miao F, Ross CJ, van Eck M, Hayden MR (2006). Elevated plasma triglyceride levels precede amyloid deposition in Alzheimer's disease mouse models with abundant A beta in plasma. Neurobiol Dis.

[B28] Takechi R, Galloway S, Pallebage-Gamarallage MMS, Wellington CL, Johnsen RD, Dhaliwal SS, Mamo JCL (2009). Differential effects of dietary fatty acids on the cerebral distribution of plasma derived apo B lipoproteins with amyloid-beta. Br J Nutr.

[B29] Takechi R, Galloway S, Pallebage-Gamarallage M, Wellington C, Johnsen R, Mamo JC (2009). Three-dimensional colocalization analysis of plasma-derived apolipoprotein B with amyloid plaques in APP/PS1 transgenic mice. Histochem Cell Biol.

